# Strategies to reduce uric acid through gut microbiota intervention

**DOI:** 10.3389/fmicb.2025.1654152

**Published:** 2025-08-29

**Authors:** Yueying Cui, Peiyu An, Feng Li, Fengsen Duan, Zusong Mei, Qiao Ye, Guangyun Wang, Haitao Zhang, Yuan Luo

**Affiliations:** ^1^Laboratory of Clinical Medicine, Air Force Medical Center, Air Force Medical University, PLA, Beijing, China; ^2^Department of Aeromedical Support & Flight Safety, Air Force Medical Center, Air Force Medical University, PLA, Beijing, China; ^3^Department of Cardiovascular Medicine, Air Force Medical Center, Air Force Medical University, PLA, Beijing, China

**Keywords:** hyperuricemia, gut microbiota, uric acid-lowering mechanism, probiotics, fecal microbiota transplantation

## Abstract

Hyperuricaemia (HUA) is a metabolic disorder resulting from the dysregulation of purine metabolism. It is closely associated with gout and various metabolic syndromes, representing an increasing global public health challenge. Current treatment approaches for HUA and gout generally involve the lifelong administration of urate-lowering agents to maintain optimal serum urate concentrations. However, poor patient adherence, often due to potential hepatorenal toxicity, frequently leads to disease relapse. Recent evidence indicates that the gut microbiota plays a significant role in maintaining urate homeostasis through multiple mechanisms, including the modulation of purine metabolism, urate catabolism and excretion, regulation of inflammatory responses, and preservation of intestinal barrier integrity. These findings highlight the gut microbiota as a promising novel therapeutic target. This review synthesizes recent progress in three key areas: (1) the relationship between the gut microbiota and HUA; (2) microbial mechanisms underlying urate-lowering effects, such as microbial purine and urate metabolism, regulation of urate transporters like ABCG2, and production of anti-inflammatory metabolites; and (3) microbiota-based therapeutic interventions, including probiotics, engineered bacterial strains, fecal microbiota transplantation, and pharmabiotic strategies. Additionally, we explore the translational potential of microbiota modulation in clinical settings and outline directions for future research. By integrating mechanistic understanding with therapeutic innovation, this review offers researchers and clinicians a comprehensive framework for advancing microbiota-targeted approaches in the management of hyperuricaemia.

## 1 Introduction

Hyperuricaemia (HUA) is a metabolic disorder resulting from dysfunction in purine metabolism. It is characterized by elevated serum uric acid (SUA) levels. In contrast, gout is a condition in which blood uric acid levels exceed the physiological solubility limit in blood or tissue fluids, leading to the formation and deposition of sodium urate crystals in local joints. This process triggers an inflammatory response and tissue damage (Estiverne et al., 2020; Mandal and Mount, 2015; Zhu et al., 2021). Due to improvements in modern living standards and lifestyle, the global incidence rates of HUA and gout have increased annually and are trending toward younger populations. These conditions have become the fourth highest in incidence after diabetes, hypertension, and hyperlipidemia, and their health impacts are increasingly pronounced. HUA and gout represent a continuous and chronic pathophysiological process characterized by significant clinical heterogeneity. Both conditions are independent risk factors for chronic kidney disease, hypertension, cardiovascular and cerebrovascular diseases, and diabetes mellitus. They are also independent predictors of premature mortality. Prolonged HUA can also lead to atherosclerosis, increasing the risk of cardiovascular diseases. Traditional treatments for HUA primarily involve medication and dietary and lifestyle interventions. Commonly used drugs are mainly categorized into two groups: those that inhibit uric acid production (e.g., allopurinol and febuxostat) and those that promote uric acid excretion (e.g., benzbromarone and probenecid). However, long-term use of these medications increases the risk of side effects in the liver and kidneys. Therefore, poor compliance is an issue. The treatment of gout necessitates a dual approach, combining anti-inflammatory therapy during the acute phase with long-term urate-lowering treatments. Patients must be educated on the importance of lifelong management, with particular emphasis on the control of comorbidities.

Recent studies have demonstrated that the gut microbiota plays a critical role in maintaining urate homeostasis through multiple mechanisms, including purine metabolism, urate excretion, regulation of inflammation, and preservation of intestinal barrier integrity. These findings highlight the gut microbiota as a promising therapeutic target for the management of hyperuricemia (HUA) and gout. This review synthesizes the most recent progress in gut microbiota-mediated urate reduction, emphasizing the relationship between the gut microbiota and HUA, elucidating the underlying mechanisms by which the gut microbiota modulate urate levels, and exploring current research and clinical applications in this area. By providing a comprehensive overview, this article aims to support researchers and clinicians in advancing the translational potential of gut microbiota-based interventions for urate reduction.

## 2 Relationship between HUA and intestinal microbiota

### 2.1 Pathophysiological basis of HUA

**Figure 1:**
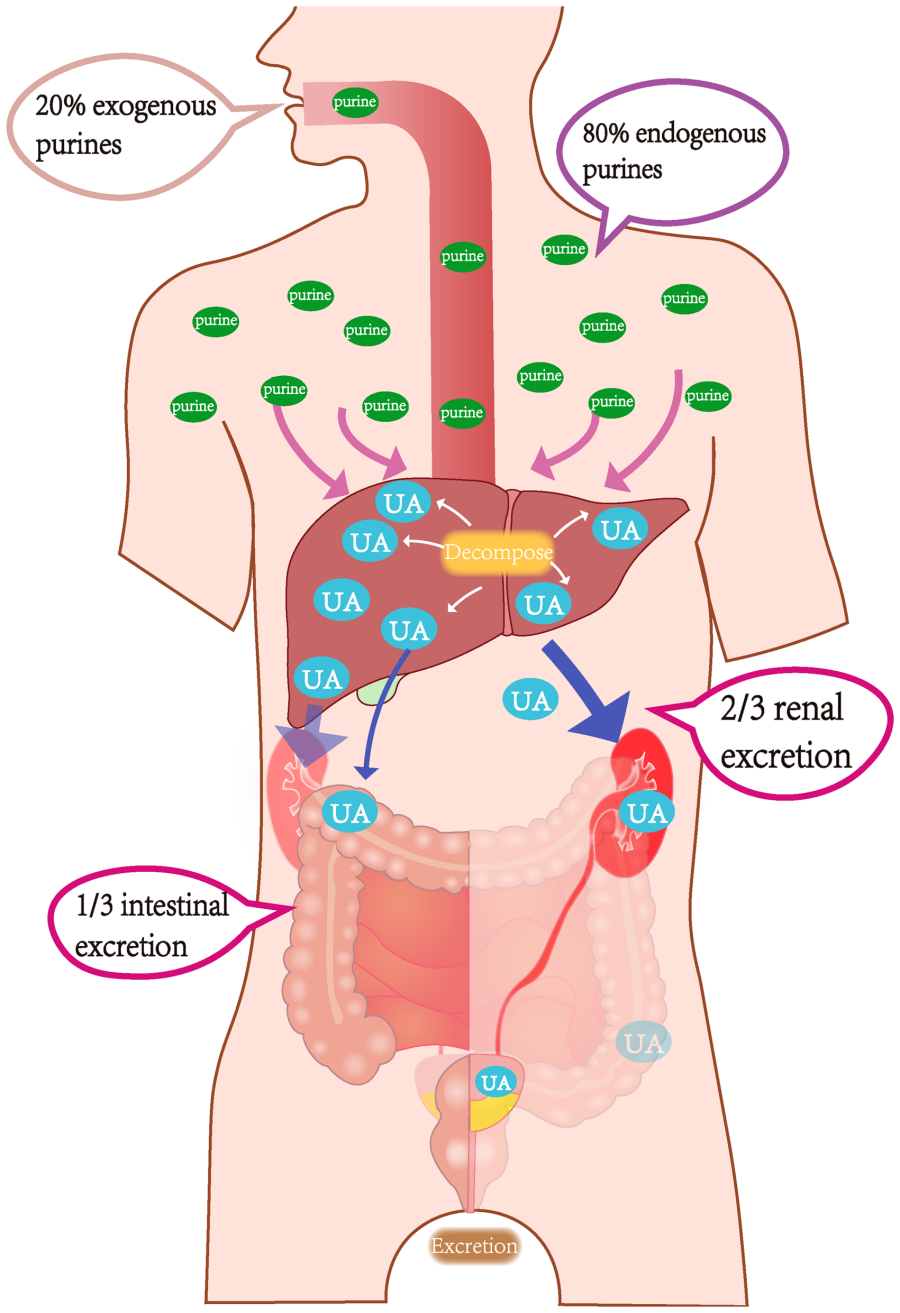
Schematic diagram of uric acid metabolism pathways in the body.

Uric acid is the final metabolite of purine metabolism. Due to a lack of uricase, humans cannot further break down uric acid, which is primarily excreted through the kidneys and intestines; these two excretion pathways account for approximately 2/3 and 1/3 of uric acid secretion, respectively. Under normal circumstances, approximately 80% of purine nucleotides are metabolized by human cells, while only 20% originate from food (Figure 1). The body maintains uric acid levels within a normal range by dynamically regulating the intake (production) and output (excretion) of uric acid. Therefore, the root causes of HUA are primarily due to two mechanisms. First, elevated uric acid levels may be due to either ingestion of a high purine diet or increased uric acid production resulting from abnormal purine metabolism or tumor lysis syndrome. Second, various kidney diseases, medication interference, or excessive organic acid production that suppresses uric acid excretion may also be a cause (Xu et al., 2016).

The primary cause of excessive uric acid production is driven by abnormal endogenous purine nucleotide metabolism. Sources of purines include exogenous high-purine diets (e.g., red meat and seafood) and endogenous cellular metabolic products (e.g., nucleic acid breakdown) (El and Tallima, 2017). The primary triggers include abnormalities in key enzyme function (e.g., hyperactivity of xanthine oxidase (XO), which accelerates the conversion of hypoxanthine to uric acid (Maesaka and Fishbane, 1998), overactivation of phosphoribosylpyrophosphate synthase (PRPS), or a deficiency in hypoxanthine-guanine phosphoribosyltransferase (HGPRT), as occurs in Lesch–Nyhan syndrome). The result is an abnormal increase in *de novo* purine synthesis (Merriman and Dalbeth, 2011). Additionally, fructose metabolism consumes ATP, generating substantial amounts of AMP, which indirectly promotes uric acid synthesis. Insufficient excretion is closely associated with abnormalities in kidney and intestinal function. Renal excretion is a critical step in SUA regulation. This is primarily mediated by various molecules expressed in the proximal tubule (Dalbeth and Merriman, 2009), such as the overactivation of URAT1 on the apical membrane and GLUT9 on the basal membrane of the proximal convoluted tubule, which leads to an increase in uric acid reabsorption (Eriksson and Lindblom, 1993). Mutations in the ABCG2 gene (e.g., Q141K) weaken uric acid secretion in the intestine and kidneys (Ruiz et al., 1989). In terms of intestinal excretion, specific bacterial flora (e.g., *Escherichia coli*, Lactobacillus, and Pseudomonas) degrade uric acid to allantoin through the secretion of uricase and allantoinase. However, patients with HUA often suffer from dysbiosis of the intestinal microbiota (Gassner and Tassava, 1997), which is characterized by a decrease in the abundance of probiotics and the proliferation of conditional pathogens (e.g., Bacteroidetes). This results in reduced intestinal uricase activity and a diminished capacity for uric acid degradation. Concurrently, the reduction of short-chain fatty acids (SCFAs) (e.g., butyrate) from microbial metabolites weakens intestinal mucosal barrier function and inhibits ABCG2-mediated uric acid excretion in the intestine (Xu et al., 2024).

### 2.2 Impact of microbiota-host co-metabolism on uric acid homeostasis

Microbiota-host co-metabolism maintains uric acid homeostasis through multi-dimensional regulatory mechanisms. The core functions include uric acid degradation, excretion regulation, and systemic inflammation suppression (Wang J. et al., 2022; Wang P. et al., 2022; Wang Z. et al., 2022). This reduces endotoxin (LPS) entry into the bloodstream and the ensuing inflammatory response, ultimately inhibiting disturbances in uric acid metabolism (Liu et al., 2023; Xu et al., 2024). Metabolomics analysis revealed a significant reduction in fecal butyrate levels in HUA patients. This metabolite regulates uric acid homeostasis through a dual mechanism: by inhibiting xanthine oxidase (XOD) activity to reduce uric acid production and upregulating the expression of the ABCG2 transporter in the kidneys and intestine to enhance uric acid excretion (Krautkramer et al., 2021; Xu et al., 2024). Metagenomic data have further indicated that in the early stages of HUA, the abundance of uric acid degradation gene clusters (e.g., uricase and allantoinase) in the gut microbiota decreased 70%, while the abundance of purine uptake genes increased 1.5-fold. This suggests that microbial dysbiosis precedes clinical abnormalities in uric acid levels (Feng et al., 2024; Xu et al., 2024; Yueqi Wang, 2022). The interaction between microbial metabolites and host signaling pathways plays a crucial role in uric acid regulation. For example, butyrate enhanced antioxidant capacity by activating the Nrf2 pathway and inhibited the NF-κB-mediated inflammatory response, thereby alleviating renal tubular damage (Liu et al., 2023; Xu et al., 2024). Additionally, probiotics such as Lactobacillus can secrete uricase, which directly breaks down intestinal uric acid. Probiotics also upregulate the expression of OAT1 and ABCG2 in the kidneys through modulation of the MAPK/NF-κB pathway, thereby promoting uric acid excretion (Liu et al., 2023; Xu et al., 2024; Yueqi Wang, 2022). Fecal microbiota transplantation (FMT) experiments also revealed that HUA microbiota can exacerbate kidney damage by activating the NLRP3 inflammasome, where supplementation with *Parabacteroides distasonis* significantly reduced SUA levels. This mechanism involved ROS scavenging and vascular endothelial repair (Bian et al., 2024; Krautkramer et al., 2021). Clinical intervention studies have demonstrated the therapeutic potential of targeting microbiota-host co-metabolism. For example, the traditional Chinese medicine (TCM) formula Guizhi Shaoyao Zhimu Decoction (GSZD) increased the abundance of Lactobacillus and Ruminococcaceae, restored glycerophospholipid metabolism and the alanine pathway, and significantly reduced inflammatory cytokine levels (Cheng et al., 2024). Additionally, folic acid and zinc inhibited xanthine oxidase activity by modulating microbial community structure, thereby increasing uric acid degradation by 56%. These findings provide a scientific basis for the development of precision intervention strategies based on microbial metabolic reprogramming (Liu et al., 2023; Xu et al., 2024; Yueqi Wang, 2022).

## 3 Mechanisms of the gut microbiota in uric acid reduction

### 3.1 Direct regulation of uric acid metabolism enzyme activity

The gut microbiota plays a central role in lowering uric acid levels by directly regulating the activity of enzymes involved in uric acid metabolism. This occurs through a multi-layered process of enzyme activity inhibition and metabolic pathway modulation. Specific enzyme inhibition by probiotic strains is a primary mechanism. For example, *Lactobacillus paracasei X11* completely degraded purine nucleotides within 30 min, significantly inhibiting hepatic xanthine oxidase (XOD) activity. This led to a 52.45% reduction in SUA levels in hyperuricemic mice and downregulation of the renal urate reabsorption proteins URAT1 and GLUT9 (Hussain et al., 2024). Additionally, *Limosilactobacillus reuteri HCS02-001* inhibited hepatic XOD activity via the TLR4/MyD88/NF-κB pathway and upregulated intestinal ABCG2 expression. The metabolic byproducts of which can induce fecal xanthine dehydrogenase and urease activity, accelerating uric acid decomposition (Zhang et al., 2024). The targeted regulation of natural compounds and TCM components can further enhance this mechanism. For example, 24 small molecules from guaijaverin directly bound to the active site of XOD, inhibiting its catalytic function and restoring the amino acid metabolic function of gut microbiota (Ji et al., 2024). Rare ginsenosides from ginseng were shown to regulate gut microbial diversity, suppress XOD activity in the serum and liver, restore renal antioxidant enzyme (SOD and GSH) activity, and reduce damage from oxidative stress (Chen et al., 2024). Salinomycin promoted NRF2 nuclear translocation to inhibit XOD activity. This resulted in the enrichment of SCFA-producing bacteria, thereby improving renal function in a model of hyperuricemic nephropathy (Guo et al., 2021). Furthermore, inulin achieved reduction in SUA by increasing the abundance of SCFA-producing bacteria, inhibiting hepatic XOD activity, and upregulating ABCG2 expression (Zou et al., 2024). In addition, the synergistic effects of microbial metabolites should not be overlooked. *Lactiplantibacillus plantarum X7022* degraded xanthine, guanine, and adenine through the purine assimilation pathway; inhibited XOD activity; achieved reduction in SUA; restored gut microbial balance; and increased SCFA levels, further inhibiting inflammatory pathways (Zhou et al., 2023). These studies collectively indicate that gut microbiota and their metabolic by-products reduce uric acid generation at its source by directly targeting key enzymes such as XOD and ADA. They also work in concert with the modulation of microbial structure and host signaling pathways (e.g., NRF2 and TLR4/NF-κB) to maintain uric acid homeostasis.

### 3.2 Regulation of the urate transporter network

Recent studies have demonstrated a pivotal role of the gut microbiota in urate excretion through modulation of urate transporter expression. Several studies have confirmed that regulating the gut microbiota ameliorated HUA by significantly affecting the function of transporters such as ABCG2, OAT1, and URAT1 (Xu et al., 2025). Probiotics reshaped gut microbiota structure by increasing the abundance of beneficial bacteria, repairing intestinal barrier integrity and upregulating colon ABCG2 protein expression. This was shown to promote urate excretion and reduce serum urate levels by more than 60% (Fang et al., 2024). Similarly, mulberry extract was shown to regulate bacterial populations. This led to inhibition of the URAT1 reabsorption channel in the kidneys and simultaneously activated the excretion function of ABCG2, achieving a dual-pathway reduction in SUA levels (Fu et al., 2024b). Additionally, metabolic products of the gut microbiota and SCFAs can directly modulate liver XOD activity and kidney transporter expression by activating the aryl hydrocarbon receptor pathway. One study showed that coffee leaf tea extract enhanced fecal SCFA levels by enriching SCFA-producing bacteria, suppressed the release of inflammatory factors, and increased urate excretion by downregulating GLUT9 while upregulating OAT3 and ABCG2 expression (Zhou et al., 2023). Numerous studies have collectively revealed that the gut microbiota dynamically regulates the urate transporter network through the metabolite-host signaling axis. This provides a theoretical basis for the development of novel targeted therapeutic strategies using the microbiota in the treatment of HUA.

### 3.3 Inflammation and immune regulation

The gut microbiota plays a crucial role in lowering uric acid levels by modulating inflammatory and immune pathways. This involves multi-layered anti-inflammatory effects and immune homeostasis restoration. Probiotics regulate urate metabolism by inhibiting pro-inflammatory factors and modulating key signaling pathways. In hyperuricemic mice, *L. paracasei X11* significantly inhibited hepatic xanthine oxidase (XOD) activity by degrading purine nucleotides, leading to reduced SUA levels. This was accompanied by downregulation of the renal urate reabsorption proteins URAT1 and GLUT9, suppression of the inflammatory factor IL-1β, and restoration of the Bacteroidetes/Firmicutes ratio in the gut microbiota. This resulted in an overall decrease in systemic inflammation (Cao et al., 2022). Similarly, *Limosilactobacillus reuteri HCS02-001* inhibited hepatic XOD activity via the TLR4/MyD88/NF-κB pathway. It upregulated intestinal ABCG2 expression, reduced SUA levels, enhanced fecal xanthine dehydrogenase and allantoinase activity, and accelerated uric acid degradation. A concurrent decrease in pro-inflammatory cytokine levels was also observed in the liver (Copur et al., 2022).

The immunomodulatory effects of SCFAs have also been documented. For example, inulin has been shown to enrich SCFA-producing bacteria, increase the intestinal concentration of butyrate and propionate, repair tight junction proteins, and reduce serum LPS and inflammatory cytokines. This led to inhibition of NF-κB-mediated in responses and downregulation of hepatic XOD activity, ultimately lowering uric acid levels (Zhao et al., 2022). Salinomycin inhibited oxidative stress through activation of the NRF2 pathway, reduced renal fibrosis and the expression of IL-1β and TNF-*α*, and promoted the proliferation of SCFA-producing bacteria. This led to improved renal function in hyperuricemic mice (Wang P. et al., 2022). Natural compounds can regulate inflammatory pathways by modulating interactions between the microbiota and the immune system. For example, 24 small molecules in guaijaverin were shown to directly bind to the active site of XOD, inhibiting its catalytic function and decreasing SUA levels. The small molecules also restored the pyruvate fermentation function of the gut microbiota, reducing amino acid metabolic disorders and indirectly suppressing the activation of the NLRP3 inflammasome (Wang Z. et al., 2024). Rare ginsenosides have been shown to regulate gut microbial diversity, inhibit serum and liver XOD activity, restore kidney SOD and GSH antioxidant enzyme activity, reduce MDA accumulation, decrease IL-1β production, and modulate Th17/Treg balance, ultimately alleviating kidney injury. This was achieved through the enrichment of *Lactobacillus* and *Akkermansia* (Lv et al., 2023).

Direct interaction between microbial metabolism and immune cells: In the animal model of hyperuricemia (HUA), intestinal dysbiosis leads to an increase in the proportion of Th17 cells and a decrease in Treg cells (Wang J. et al., 2022). Probiotic *Lactiplantibacillus pentosus* P2020 inhibits renal inflammation by downregulating the MAPK and TNF-α pathways, while upregulating ABCG2 and OAT1 expression to promote urate excretion (Wang et al., 2023). Additionally, metabolites derived from the gut microbiota activated the G protein-coupled receptor GPR43/41, inhibited NLRP3 inflammasome activation, reduced IL-18 release, repaired intestinal barrier function, and decreased systemic inflammation caused by endotoxins entering the bloodstream (Yang et al., 2023). In summary, the gut microbiota can alleviate inflammatory damage due to HUA through multiple mechanisms including inhibition of XOD activity, regulation of the NF-κB/NLRP3 inflammatory pathway, balancing of Th17/Treg cells, and enhancing immune homeostasis mediated by SCFAs (Lv et al., 2023; Mamun et al., 2025; Shirvani-Rad et al., 2023; Wang P. et al., 2022). These mechanisms provide a theoretical foundation for precise interventions targeting the microbiota-immune axis.

### 3.4 Gut microecological remodeling

The gut microbiota governs uric acid flux by rewiring its own ecosystem, and three mechanistic nodes now account for this control. First, *Lactobacillus reuteri* HCS02-001 secretes nucleoside hydrolase, degrading intestinal purine nucleosides, curbing their absorption, and suppressing hepatic xanthine oxidase (XOD) (Hussain et al., 2024; Wang Q. et al., 2024). Concomitantly, ABCG2-mediated renal excretion rises while GLUT9-driven reabsorption falls, forging a bidirectional urate-regulatory axis. Second, *L. plantarum* SQ001 catabolizes xanthine and adenine via its purine-assimilation pathway, directly lowering serum uric acid (SUA) (Fu et al., 2024b; Xu et al., 2025).

Prebiotics and traditional Chinese medicine (TCM) amplify these effects. Inulin enriches SCFA-producers, elevates butyrate, restores tight-junction proteins, and curtails LPS and pro-inflammatory cytokines; the resulting drop in systemic inflammation feeds back to suppress XOD activity (Zou et al., 2024). Twenty-four small molecules in FangyuKangsu granules dock into XOD’s catalytic pocket, block its activity, and redirect purine flux toward SCFA fermentation while reshaping the microbiota (Ankli, 2024).

Metabolite signaling completes the circuit. Butyrate inhibits NLRP3 inflammasome activation and IL-18 release through G-protein-coupled receptors and represses TLR4/NF-κB via the aryl hydrocarbon receptor, indirectly lowering XOD (Ji et al., 2024; Sun et al., 2025; Zhou et al., 2023). Rare ginsenosides (Rg3 and Rg5) modulate sphingolipid and pyrimidine metabolism, expand Lactobacillus and Akkermansia, and markedly inhibit serum XOD (Guo et al., 2021).

Clinically, dysbiosis typifies hyperuricaemia: diversity falls, pathogens surge, and SCFA-producing commensals decline, eroding the intestinal barrier and urate excretion (Sun et al., 2024; Xu et al., 2021). Fecal microbiota transplantation and post-biotic or herbal interventions reprogram tryptophan metabolism, recapitulate XOD inhibition, and restore functional microbial modules (Chen et al., 2024; Sun et al., 2024, 2025). High-fat-diet-induced dysbiosis further aggravates uric-acid imbalance via PI3K/AKT/mTOR activation, whereas curcumin reverses this trajectory by suppressing pathobionts and boosting Lactobacillus and Ruminococcaceae (Hussain et al., 2024; Xu et al., 2021).

Therefore, microbiota remodeling offers a precision strategy for hyperuricaemia: by concomitantly reshaping community composition, metabolic output, and host signaling, it targets uric-acid homeostasis at multiple checkpoints—an approach especially valuable for patients with renal impairment or intolerance to conventional drugs.

## 4 Strategies for decreasing uric acid levels through the gut microbiota

### 4.1 Application of probiotics

A coherent mechanistic arc now links specific probiotic strains to reduced SUA. *In vitro* and animal data demonstrate selected strains [including Lactobacillus CICC 6074 and 20,292 from PSFA studies (Li et al., 2024)] suppress hepatic xanthine oxidase activity (as evidenced by PSFA’s xanthine oxidase inhibition) (Sun et al., 2024), up-regulate ABCG2 and SLC2A9 transporters (Sun et al., 2024), and enrich SCFAs that curb NLRP3 activation via FFAR signaling, attenuating renal and colonic inflammation (Han et al., 2023; Wang Q. et al., 2024) (consistent with PSFA’s reduction in creatinine/urea levels and amelioration of kidney damage). Concomitantly, tryptophan is funneled to indole-3-propionic acid, an AhR agonist that suppresses TLR4/MyD88/NF-κB while tightening the gut barrier (Han et al., 2023; Wang Q. et al., 2024) (aligning with PSFA-induced enrichment of Lachnospiraceae_NK4A136_group and Faecalibaculum).

Human trials corroborate these effects: randomized studies (Hussain et al., 2024; Sun et al., 2024) report 10–15% SUA reductions with Lactobacillus supplementation (paralleling PSFA’s uric acid-lowering effects), accompanied by decreased fecal xanthine oxidase activity and elevated ABCG2 expression, while metagenomic analyses document increased butyrate production and reduced IL-1β (further supported by PSFA’s gut microbiota remodeling outcomes) (Wang Z. et al., 2024).

Yet a third trial with genetically similar isolates from fermented foods reports no SUA change despite intact barrier repair (Wang Q. et al., 2024). The discord points to unmonitored safety variables—strain persistence, immune imprinting, or host-microbiome context (Han et al., 2023)—underscoring the need for extended follow-ups and strain-specific risk profiling to translate mechanism into consistent therapy.

### 4.2 Prebiotics and synbiotics

Prebiotics and synbiotics remodel the gut microbiota to accelerate uric acid disposal.

Mechanistically, inulin and fructooligosaccharides act as selective carbon sources that expand purine-fermenting taxa and up-regulate uricase and allantoinase, trimming serum uric acid (SUA) by ~10% (He et al., 2022; Lai et al., 2019). The resulting SCFA surge simultaneously tightens the epithelial barrier and quenches uric-acid–driven renal inflammation (He et al., 2022; Singh et al., 2024). And fucoidan alleviates hyperuricemia via dual inhibition of uric acid production (XOD/ADA suppression) and promotion of excretion (ABCG2 upregulation/GLUT9 downregulation), while restoring gut microbiota diversity and enrichment of beneficial taxa, offering a therapeutic alternative for drug-intolerant patients (Wang et al., 2025). Lophatherum gracile directly suppresses uric acid production by inhibiting xanthine oxidase and adenosine deaminase, while blocking renal reabsorption via GLUT9 downregulation and promoting excretion through ABCG2 upregulation (Lu et al., 2025).

Evidence from clinical settings confirms this cascade. In gout patients already receiving allopurinol, a synbiotic pairing of Lactobacillus plus prebiotic fiber lowered SUA and CRP, shifted the Firmicutes/Bacteroidetes ratio toward purine degraders, and—via metatranscriptomic analysis—elevated intestinal ABCG2 and GLUT9 expression, thereby restoring the gut-liver-kidney urate axis (Kondratiuk et al., 2020; Lai et al., 2019).

Taken together, these data position prebiotics and synbiotics as safe, evidence-based adjuncts for hyperuricemia, particularly in patients with renal impairment or intolerance to standard therapies (Singh et al., 2024). Emerging Gut Microbiome-Tailored Urate Therapy (GM-TUT) leverages this mechanistic clarity: baseline metagenomic profiling of purine catabolism, SCFA potential, and transporter expression guides bespoke prebiotic/synbiotic formulations that maximize bacterial engraftment, amplify uric acid catabolism, and minimize non-response.

### 4.3 Dietary intervention: targeted modulation of microbial composition

The Mediterranean and Dietary Approaches to Stop Hypertension (DASH) diets have been shown to reduce SUA levels by optimizing microbial composition (Sun et al., 2024). The Mediterranean diet, which is centered around whole grains, olive oil, fruits, vegetables, and nuts, features high levels of antioxidants such as polyphenols. These compounds inhibit the proliferation of pro-inflammatory bacteria while promoting the growth of SCFA-producing microorganisms. This increase in butyrate levels suppresses the activity of xanthine oxidase (XO) and reduces uric acid synthesis. Research indicates that a one-month Mediterranean diet intervention reduced SUA levels in hyperuricemic patients from 9.12 mg/dL to 6.92 mg/dL and decreased systemic inflammatory factors, ameliorating purine metabolism disorders (Chrysohoou et al., 2011; Sun et al., 2024; Yokose et al., 2021). The DASH diet, characterized by its low-purine and high-fiber properties, has been shown to reduce the abundance of pathogenic bacteria. Among patients with baseline SUA ≥ 7 mg/dL, a DASH diet intervention caused a significant decrease in SUA. This mechanism was associated with the suppression of the LPS-induced TLR4/NF-κB inflammatory pathway (Cardoso-Jaime et al., 2022; Juraschek et al., 2016; Rai et al., 2017; Song et al., 2021).

Polyphenols such as sweet potato anthocyanins and caffeic acid can directly inhibit XO activity. Studies in hyperuricemic mice showed that anthocyanins formed hydrogen bonds with the active site of XO, blocking substrate binding and reducing SUA. Simultaneously, expression of the renal excretion proteins ABCG2 and OAT1 were upregulated, promoting uric acid excretion (Li et al., 2021; Zhang et al., 2019). The alkaloid berberine reduced uric acid reabsorption by downregulating URAT1 and GLUT9 and inhibiting the NLRP3 inflammasome, alleviating renal inflammatory damage (Li et al., 2021). Additionally, a low-sugar diet (such as restricting fructose intake) reduced hepatic ATP depletion and decreased purine catabolism, thereby lowering endogenous uric acid production (Caliceti et al., 2017). Studies have also shown that a high-fiber diet can enhance the ability of the gut microbiota to degrade uric acid (Vande et al., 1994). Clostridium and Pseudomonas metabolize uric acid into the more soluble allantoin, facilitating intestinal excretion (Makki et al., 2018; Mendez-Salazar and Martinez-Nava, 2022). Furthermore, a low-fat diet decreased LPS release, improved gut barrier function, and decreased systemic inflammation-induced stimulation of XO activity (Shen et al., 2014). These dietary strategies offer a safe and sustainable approach to managing HUA by reshaping microbial community structure, enhancing the anti-inflammatory effects of SCFAs, and optimizing purine metabolism.

### 4.4 Innovative applications of FMT

The FMT is an innovative therapeutic method that systematically reshapes host gut microecology by directly introducing microbial communities from healthy donors or those subjected to specific interventions to regulate uric acid metabolism (Wang et al., 2019). The core principle of FMT involves the transfer of microbial communities and their metabolic products to ameliorate uric acid synthesis-excretion balance and the disruption of inflammatory pathways (Song et al., 2023). One study showed that in a goose model of HUA, transplantation of gut microbiota pre-treated with probiotics significantly enhanced intestinal nucleotide degradation through purine degradation enzymes. This enhancement led to a reduction in SUA levels via the “gut-liver-kidney” axis (Fu et al., 2024a). Furthermore, FMT has demonstrated the ability to correct microbial community dysbiosis and metabolic deficiencies in HUA patients and animal models, restoring systemic metabolic homeostasis. Following treatment with oleanolic acid (OA), the gut microbiota of mice was transplanted via FMT into HUA recipients, leading to a significant upregulation of intestinal urate transporters, such as ABCG2 and URAT1. This enhancement promoted uric acid excretion and repaired the intestinal barrier to reduce endotoxin translocation, alleviating renal inflammation (Zhang et al., 2025). FMT validation of the mechanism of the TCM compound Quzhuo Tongbi Decoction (QZTBD) confirmed that microbiota remodeling activated the PI3K-AKT–mTOR pathway, regulated Th17/Treg immune balance, and suppressed the release of inflammatory factors, such as IL-1β (Song et al., 2023). In clinical practice, the combined treatment of FMT with the Chinese medicine QYHT for HUA-related erectile dysfunction modulated microbiota metabolites to inhibit the activation of the NLRP3 inflammasome, thereby improving oxidative stress and sexual function indicators (Ge et al., 2025; Song et al., 2023). These studies highlight that FMT can not only directly restore microbial diversity but also regulate the host metabolic network through multiple targets. These studies provide precise and sustainable intervention strategies for refractory HUA and its complications.

The FMT still faces a chain of unresolved issues that propagate directly into clinical uncertainty. Because each protocol differs in donor selection, stool processing, and administration route, treatment heterogeneity undermines both cross-trial comparability and patient-level response prediction. This heterogeneity, in turn, amplifies five persistent risks: (1) acute recipient harm, (2) sub-optimal or unknown dosing, (3) uncontrolled confounding from diet, environment, and co-medications, (4) transient rather than durable remission, and (5) poorly defined recipient-specific success determinants. Consequently, existing studies—typically limited to 8–12 weeks of follow-up—cannot disentangle these confounders or capture long-term efficacy and safety signals. Therefore, extended, harmonized trials that systematically manipulate and monitor each variable are indispensable before FMT can move from experimental rescue therapy to routine care (Yadegar et al., 2024).

### 4.5 Multi-targeted regulation using TCM

**Figure 2:**
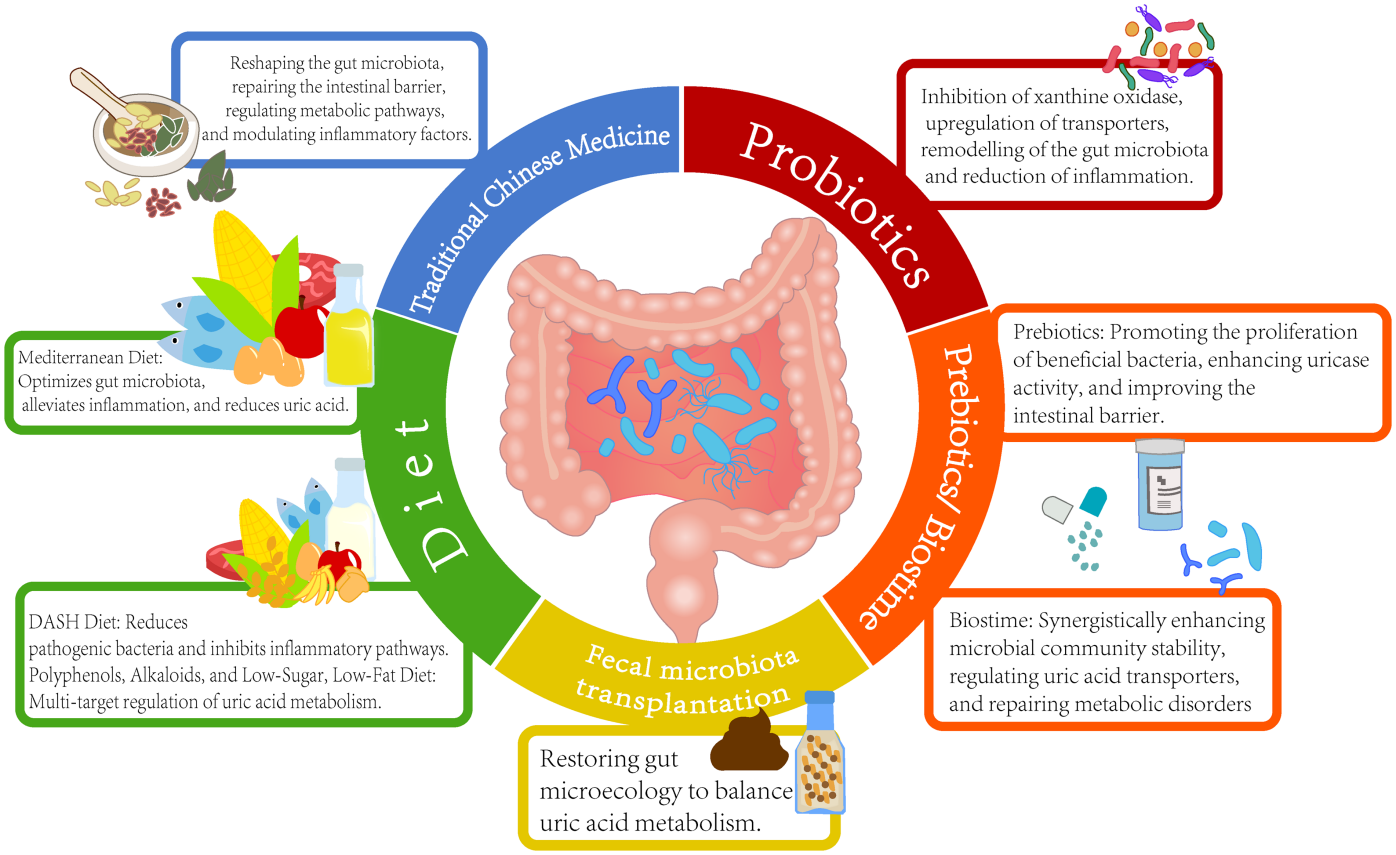
Uric acid reduction strategies based on gut microbiota.

Among the various strategies for uric acid reduction, TCM demonstrates the unique advantage of regulating the microbiota-host metabolic network through multiple targets. Research indicates that various TCM formulas, such as GSZD, QZTBD, and *Cichorium intybus* formula (CILF), can reshape gut microbiota structure, repair intestinal barrier function, and synergistically regulate key pathways of uric acid metabolism (Bian et al., 2024; Wang and Jin, 2024). QZTBD significantly enriched beneficial bacteria; restored Th17/Treg immune balance through the PI3K-AKT–mTOR pathway; inhibited inflammatory factors, such as IL-1β and IL-6; and simultaneously upregulated ABCG2 expression to promote urate excretion. Additionally, CILF improved renal inflammatory damage by modulating the IL-17 and TNF signaling pathways (Amatjan et al., 2023; Bian et al., 2024; Dong et al., 2024; Song et al., 2023; Zhu et al., 2023). Cat’s whiskers (CILF) reduced SUA levels, increased the number of gut uric acid-degrading bacteria, upregulated intestinal ABCG2 transporter expression, and promoted urate excretion through the gut (Zhu et al., 2023). Pharmacological analysis indicated that the active components of TCM can target and regulate key proteins, such as STAT3 and VEGFA, and influence pathways related to purine metabolism and glycerophospholipid metabolism (Cheng et al., 2023). Clinical studies have confirmed that the Yishen Huashi formula enriched beneficial bacteria in chronic kidney disease patients, reduced proteinuria, and improved lipid metabolism (Zhang et al., 2024). Additionally, TCM interventions have been shown to mediate systemic effects through the regulation of intestinal metabolites, such as SCFAs and indole derivatives. The Sanhua Decoction elevated acetic and butyric acid levels in the blood, promoted the conversion of microglia toward the anti-inflammatory M2 phenotype, and mitigated blood–brain barrier damage following stroke (Luo S. et al., 2023). FMT experiments have validated the efficacy of TCM in modulating the gut microbiome. For example, OA intervention significantly reduced uric acid levels in recipient mice post-transplantation. These findings highlight the multi-dimensional synergistic effects of TCM through the “microbiota-metabolism-immune” axis, offering a safe and multi-targeted intervention strategy for the treatment of HUA and its associated complications (Figure 2 and Table 1).

**Table 1:** Mechanisms of various methods for lowering uric acid.

Methods	Subjects	Mechanism of intervention	Reference
Probiotics	Hyperuricemia mice	Direct UA degradation and systemic modulation of gut-kidney axis	Wu et al. (2021)
Probiotics	Hyperuricemia mice	Directly degrading UA and inhibiting its synthesis; Reshaping gut microbiota to restore microbial purine-tryptophan metabolic networks; Leveraging AhR signaling to synchronize UA excretion and anti-inflammatory responses	Wang Q. et al. (2024)
Prebiotics and synbiotics	Patients with primary gout	Synbiotic group showed disease remission markers (reduced inflammation, normalized microbiota)	Kondratiuk et al. (2020)
Diet	Human	Limiting purine-rich/fructose-containing foods, enhancing antioxidant/anti-inflammatory effects, and improving metabolic parameters	Gao et al. (2021)
Fecal microbiota transplantation	Hyperuricemia mice	Drove UA metabolism regulation and inflammation suppression	Lu et al. (2021)
Fecal microbiota transplantation	Rats	The microbial taxa may associate with the occurrence of hyperuricemia	Liu et al. (2020)
Traditional Chinese Medicine	Public databases	Drug metabolism-other enzymes, Metabolic pathways, Bile secretion, Renin-angiotensin system, Renin secretion by core targets HPRT1, REN and ABCG2	Luo J. J. et al. (2023)

## 5 Summary and prospects

Gut microbiota interventions execute uric acid (UA)-lowering through four concerted mechanisms: bacterial uricase catabolizes UA into soluble allantoin; upregulated ABCG2/OAT1 transporters enhance renal/enteric excretion; suppressed URAT1/GLUT9 importers block reabsorption; and SCFA/indole derivatives quench UA-driven inflammation by inhibiting TLR4/NF-κB signaling—collectively enabling probiotics, FMT, or TCM formulations to achieve targeted UA control without organ toxicity, with patient stratification guided by microbial biomarkers (Prevotella abundance, butyrate:acetate ratios) (Li et al., 2025).

However, clinical translation faces mechanistic barriers: strain-specific purine metabolism requires mapping xanthine dehydrogenase pathways in Lactobacillus; dynamic host-microbe crosstalk demands quantification of FFAR2/3-mediated SCFA signaling to renal transporters; and limited human evidence necessitates ethnic-stratified RCTs tracking fecal purine metabolites, while biomarker personalization gaps call for AI-platforms integrating metagenomics and serum UA kinetics.

Bridging these gaps prioritizes engineered bio-therapeutics (recombinant uricase-expressing probiotics, nano-encapsulated metabolites targeting XO/GLUT9), diagnostic-stratified interventions (probiotic cocktails calibrated to Prevotella/SCFA profiles → synthetic consortia replacing hepatotoxic drugs), and cross-system validation (health-economic analyses against allopurinol, long-term safety monitoring via multi-omics registries)—ultimately positioning microbiota-directed strategies as first-line solutions for refractory hyperuricemia.

**Figure 3:**
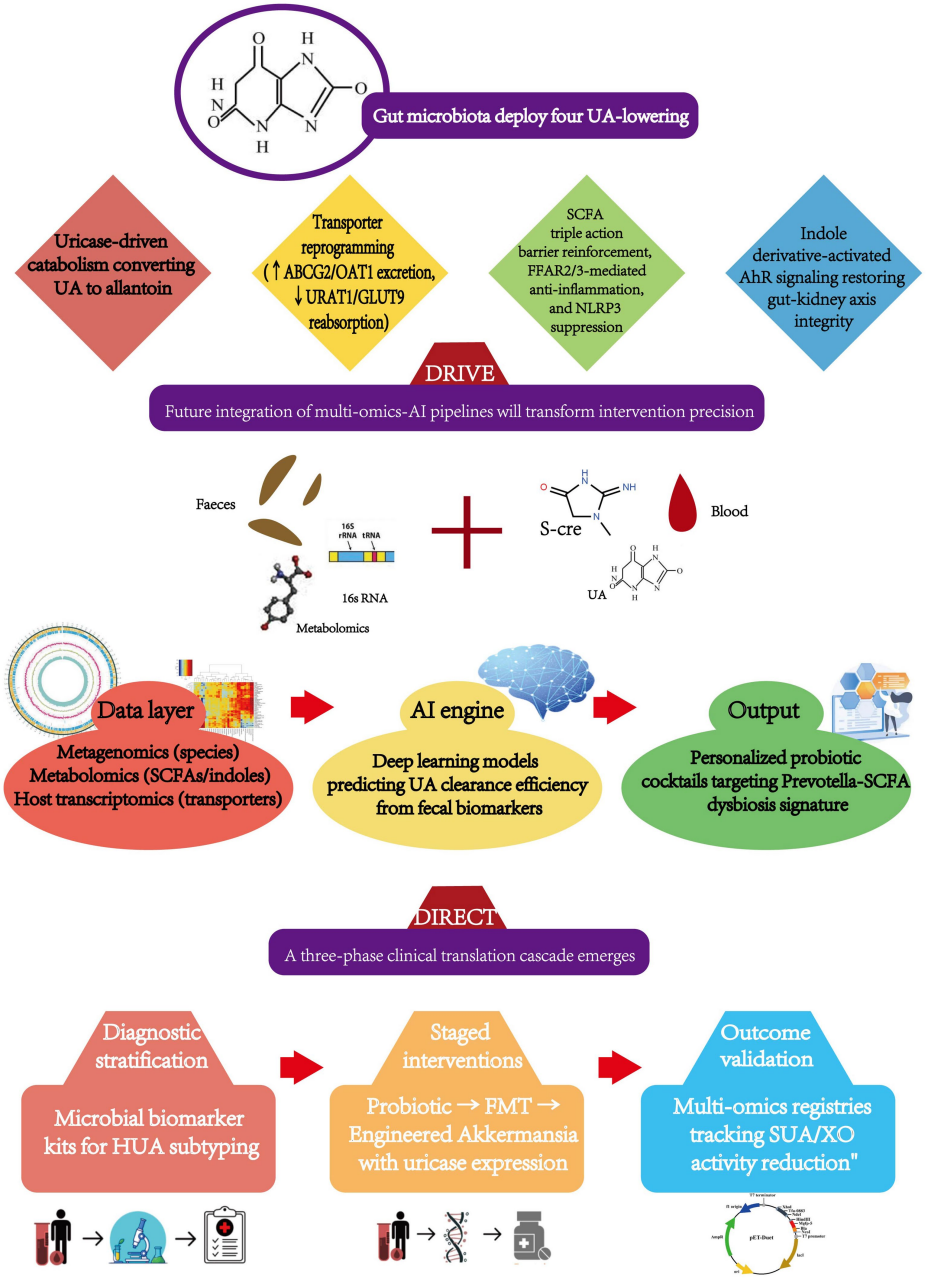
Precision modulation framework for uric acid homeostasis: gut microbiota interventions from molecular targets to AI-guided implementation.

In summary, intervention through the gut microbiota offers a novel perspective for the prevention and treatment of HUA. The integration of its multidimensional regulatory mechanisms with targeted intervention strategies holds promise for overcoming the limitations of traditional treatments. Future research is required to expand mechanistic exploration, drive clinical translation, and ultimately achieve widespread application of “microbiota precision regulation” in the treatment of metabolic diseases (Figure 3).
